# Oral Health Behavior and Lifestyle Factors among Overweight and Non-Overweight Young Adults in Europe: A Cross-Sectional Questionnaire Study

**DOI:** 10.3390/healthcare4020021

**Published:** 2016-04-06

**Authors:** Annamari Nihtila, Nicola West, Adrian Lussi, Philippe Bouchard, Livia Ottolenghi, Egita Senekola, Juan Carlos Llodra, Stephane Viennot, Denis Bourgeois

**Affiliations:** 1 Institute of Dentistry, School of Medicines, Faculty of Health Sciences, University of Eastern, P.O.B. 1627, 70211 Kuopio, Finland; annamari.nihtila@fimnet.fi; 2 Department of Public Health, Faculty of Dental Medicine, University of Lyon 1, Villeurbanne 69100, France; stephane.viennot@univ-lyon1.fr; 3 School of Oral and Dental Science, Bristol Dental School & Hospital, Bristol BS1 2LY, UK; n.x.west@bristol.ac.uk; 4 Department of Preventive, Restorative and Paediatric Dentistry, University of Bern, Bern 3012, Switzerland; adrian.lussi@zmk.unibe.ch; 5 Department of Periodontology, U.F.R. of Odontology, Paris 5-Descartes University, Paris 75006, France; phbouch@noos.fr; 6 Department of Sciences, University Sapienza, Roma 00185, Italy; Livia.Ottolenghi@uniroma1.it; 7 Riga Institute, University of Riga, Riga 1007, Latvia; esenakol@latnet.lv; 8 Juan Carlos Llodra, Department of Public Health, Faculty of Dental Medicine, University of Granada, Granada 18010, Spain; jllodra@hotmail.com

**Keywords:** overweight, young adults, oral health behavior, soft drinks, energy drinks, dietary advice, physical exercise

## Abstract

Being overweight is a risk factor for many chronic diseases including oral diseases. Our aim was to study the associations between oral health behavior, lifestyle factors and being overweight among young European adults, 2011–2012. The subjects constituted a representative sample of adult population aged 18–35 years from eight European countries participating in the Escarcel study. The participants completed a self-administered questionnaire on dietary habits, oral health behavior, smoking, exercise, height, and weight. Overweight was defined as body mass index (BMI) ≥ 25 kg/m^2^ using the World Health Organization criteria. Mean BMI was 23.2 (SD 3.48) and 24.3% of the study population were overweight. Those who were overweight drank more soft drinks (*p* = 0.005) and energy drinks (*p* = 0.006) compared with those who were non-overweight. Brushing once a day (OR 1.6; 95% CI 1.3-2.0), emergency treatment as the reason for last dental visit (OR 1.6; 95% CI 1.3–1.9) and having seven or more eating or drinking occasions daily (OR 1.4; 95% CI 1.1–1.7) were statistically significantly associated with overweight. Associations were found between oral health behavior, lifestyle and overweight. A greater awareness of the detrimental lifestyle factors including inadequate oral health habits among overweight young adults is important for all healthcare providers, including oral health care professionals.

## 1. Introduction

Being overweight is a serious and costly public health problem influencing general and oral health [1,2]. Oral diseases are among the most prevalent chronic diseases [3,4]. Globally, oral diseases affected 3.9 billion people in 2010. Untreated caries in permanent teeth was the most prevalent condition and severe periodontitis the 10th most prevalent condition according to the Global Burden of Diseases, Injuries, and Risk Factors (GBD) Study [5]. They share many common risk factors with other chronic conditions such as cardiovascular disease, type-2 diabetes, hypertension, coronary-heart diseases, certain cancers [6] and infections [7]. Health promotion strategies using the common risk factor approach have been beneficial in integrating oral health more closely to general health [8,9].

Sedentary lifestyle, and eating and drinking habits are closely associated with being overweight and oral conditions [1]. Being overweight in adolescence and in young adulthood strongly predicts the risk for obesity and metabolic syndrome in later life [10]. Overweight young adults are physically less active, eat more frequently, eat sweets every day and prefer fast food over fruit and vegetables compared with normal weight young adults [11]. In particular, rising consumption of sugary drinks has contributed greatly to the obesity epidemic [12] and sugary drinks are an important cause of dental caries [13]. Smoking increases the prevalence of periodontal disease and smokers are 2.6 to 6 times more likely to have periodontal destruction than nonsmokers [14]. On the other hand, regular consumption of dairy products seems to be beneficial for weight control [15] and for oral health [16]. Lifestyle modification is recommended as the primary treatment intervention for overweight and obese individuals [1].

There is no representative data on the relationship between being overweight and oral health habits in a population of young European adults. For this age group, it is important to target preventive measures and address common risk factors as their preventive practices and dietary patterns may be undergoing change, in order to improve general health and oral health [10]. As oral health professionals are numerous in Europe [17] and many young adults regularly visit dentist or dental hygienists [18], they are an important group to involve in reducing the amount of those who are overweight.

The hypothesis behind the present study was that poor oral health habits and poor lifestyle habits are positively associated with being overweight in the young European adult population. Thus, the purpose of the present study was to evaluate the possible relationship between oral health behavior (tooth brushing and regular dental visits), lifestyle characteristics (smoking, exercise, eating habits), and being overweight in a representative sample of the adult population aged 18–35 years in eight European countries.

## 2. Materials and Methods

This research is part of a larger study: The European Study in Non Carious Cervical Lesions (ESCARCEL). Details of the study protocol, sample calculations and participant selection have been published in 2013 [19,20].

### 2.1. Study Population

A nationally representative sample of the adult population aged 18–35 years attending general dental clinical practices from five European countries (the Czech Republic, Estonia, France, Italy and Latvia) and regionally representative from Finland, Spain and United Kingdom was obtained in 2011–2012 using a multistage, stratified sampling method. Inclusion criteria: patients aged 18–35 years in good health attending for routine dental examination, able to understand and complete the questionnaire and having a minimum of six teeth. Exclusion criteria: patients wearing an orthodontic appliance, having cervical restorations in the six eligible teeth, were taking analgesics or had undergone local oral anaesthesia in the preceding 24 hours, required antibiotic coverage for dental treatment and subjects on anticoagulants, or were suffering from bleeding disorders. The total number of participants was 3137–1719 women (54.8%) and 1418 men (45.2%). Nine participants were excluded in this study for not reporting their height and weight (one man and eight women). Mean age of women was 26.9 years and of men 28.7 years—the difference was not statistically significant. Most of the study population (69.6%) had been studying until the age of 20 years or more, or were still studying.

### 2.2. Ethics

The ethical approval was obtained from the respective Research Ethics Committees in each participating country. Oral and written informed consent was obtained from all subjects in their national language.

### 2.3. Body Mass Index (BMI) Measurement and Classification

To assess the weight status of participants, the BMI was calculated from weight (in kilograms) divided by the square of height (in square meters). The weight status was classified into two categories: Underweight or normal weight (BMI ≤ 24.9 kg/m^2^), and overweight (BMI ≥ 25 kg/m^2^) according to the World Health Organization´s International BMI Classification [21]. According to these WHO recommendations, BMI values are age-independent and the same for both sexes.

### 2.4. Questionnaire

All study participants completed a self-administrated structured questionnaire. The questionnaire was developed in English and translated into the languages of the participating countries. All translated questionnaires were linguistically validated in pilot studies. All questions were closed format questions.

This questionnaire included questions on dietary habits, smoking and exercise. Responses on dietary questions and question on smoking were made on a four-point scale: often, occasionally, rarely, never. In addition, the participants were asked questions about oral health behavior (brushing frequency, what kind of toothbrush they used, use of fluoride toothpaste, time of last dental visit and reason for the last dental visit). The participants also reported their height and weight.

### 2.5. Statistical Analyses

Data were analysed by means of IBM SPSS version 23.0 (Statistical Package for the Social Sciences) (IBM Corp, Armonk, NY, USA). The analyses included descriptive statistics giving percentages, means and standard deviation. Differences between the non-overweight and overweight young adult groups were evaluated by chi-square and Mann–Whitney tests. All of the four point scale responses were dichotomized, so that often, occasionally, rarely and never were combined. In the binary logistic regression analysis, overweight was used as the dependent variable, and the other parameters were entered individually as independent variables (adjusted for age and gender). *p* < 0.05 was considered to indicate a significant difference.

## 3. Results

### 3.1. Prevalence of Being Overweight

**Figure 1:**
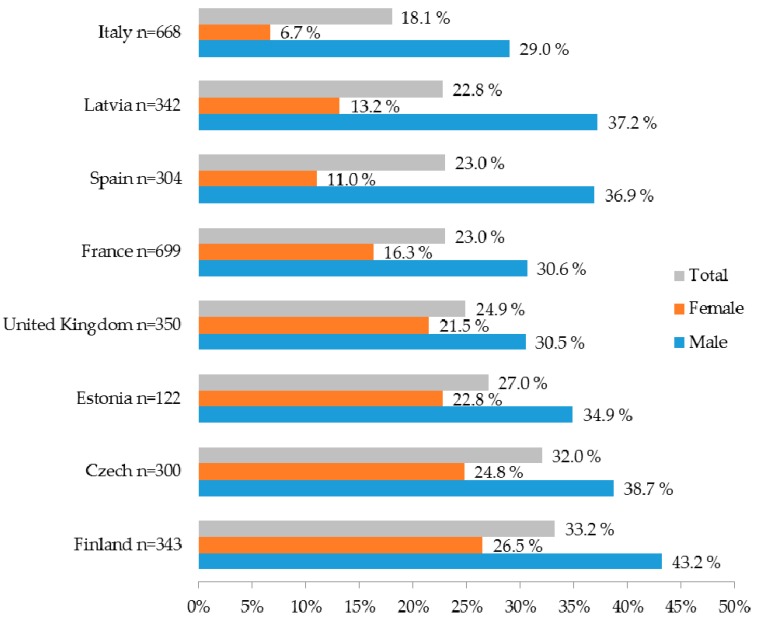
Prevalence of being overweight in 18–35 year old adults in participating countries by gender.

BMI was 23.2 (SD 3.48) and 24.3% of the study population was overweight (BMI ≥ 25). The proportion of overweight young adult population varied from 18.1 % in Italy to 33.2% in Finland (Figure 1). There were significantly more overweight males than females in all countries (*p* < 0.001). Over 40% of the Finnish young men reported being overweight.

### 3.2. Oral Health Behavior and Being Overweight

The proportion of overweight persons brushing their teeth at least twice a day (77.8%) and having had a dental visit in the past two years (81.6%) was significantly lower than in the non-overweight group (86.9%, 86.8% respectively) (Table 1). Approximately the same proportion (72%) in both groups reported using fluoride toothpaste and approximately 78% in both groups brushed their teeth with manual toothbrush. Among the overweight young adults, the reason for the last dental visit was more often emergency treatment (21.3%) than among the normal or underweight persons (13.9%) (*p* < 0.001).

### 3.3. Lifestyle Factors and Being Overweight

Lifestyle factors of overweight and non-overweight young adults are compared in Table 2. The overweight persons had significantly more often seven or more eating or drinking occasions (*p* < 0.001), drank more energy drinks (*p* = 0.006) and soft drinks (*p* = 0.005), exercised less (*p* = 0.002) and smoked more (*p* = 0.021). The mean number of eating or drinking occasions in the overweight group was 5.6 (SD 3.3) compared with 5.3 (SD 3.1) in the non-overweight group (*p* = ns).

### 3.4. Relationship of Being Overweight to Demographic, Oral Hygiene, Smoking and Dietary Factors

Binary logistic regression analysis was used to investigate whether demographic, oral hygiene, smoking or selected dietary variables were significantly associated with being overweight (Table 3). Overweight young adults had significantly (*p* < 0.001) higher odds belonging to the older age group 26–35 years (OR = 1.8, CI = 1.5–2.1) and being male (OR = 2.6, CI = 2.2–3.0). When adjusted for age and gender brushing only once a day (OR = 1.6, CI = 1.3–2.0), the last dental visit more than two years ago (OR = 1.3, CI = 1.1–1.7) and the reason for last dental visit being emergency treatment (OR = 1.6, CI = 1.3–1.9) remained significant independent variables. Those overweight were also more likely to have seven or more eating or drinking occasions daily (OR = 1.4, CI = 1.1–1.7) and exercise less than three times a week (OR = 1.3, CI = 1.1–1.5).

## 4. Discussion

The objective of this study was to describe the associations between overweight and oral health behavior and lifestyle habits in young adults in eight European countries. The main findings were that there were significant differences with regard to oral health behavior and some lifestyle habits, especially exercising less and having higher numbers of eating and drinking occasions daily between the body-weight groups.

### 4.1. Oral Health Behavior and Being Overweight

More than one-fifth of the overweight young adults reported brushing only once a day compared with 13% of the non-overweight young adults although brushing twice a day with fluoride toothpaste has been widely recommended, and it is well known that biofilm control by mechanical cleaning is a main factor in preventing periodontal disease and caries [22]. Good oral hygiene among the overweight persons should be emphasized, as there seems to be a positive association between chronic periodontal disease and being overweight and obesity [23]. Being overweight or obesity seem to be both an indirect risk factor for periodontitis, as they affect glycaemic control, and a direct risk factor because adipose tissue secretes pro-inflammatory substances that modify the periodontal tissue reaction to the dental biofilm [24]. The prevalence of severe periodontitis increases gradually with age; however, there is a steep increase between 30–40 years of age [25]. Therefore, assessing and reducing risk factors and diagnosing early signs of periodontitis in young adults is of utmost importance.

Unfortunately, the young overweight persons were more likely to report not having dental visits during the past two years and emergency treatment as the reason for their last visit compared with non-overweight persons. This is of concern as it has been stated that problem-oriented users have poorer oral health, and they are likely to have more emergency visits than regular dental attenders [26,27]. Therefore, enhancing regular dental visiting behavior among the overweight persons could markedly improve their oral health status.

### 4.2. The Use of Sugar-Sweetened Beverages

A critical problem is the rising use of non-milk extrinsic sugars called “free sugars”, mainly from two sources: sweeties and sugar-sweetened beverages (SSB) such as soft drinks, sport drinks, energy drinks, fruit drinks and flavored teas and coffees. WHO report on diet, nutrition, and the prevention of chronic diseases [13], and states that there is plenty of confirmation that these free sugars increase risk of many other diseases than dental caries, either independently or through increased risk of being overweight. For dental caries, the dietary sugars are the main cause and, without sugar consumption, the prevalence of dental caries would be insignificant [4]. However, by regularly brushing twice a day with fluoride toothpaste, brushed teeth can cope with up to six eating or drinking occasions [28]. A recent systematic review and meta-analysis [29] provided clear evidence that the use of sugar-sweetened beverages promotes weight gain both in children and adults. In this study, the overweight persons had more drinking and eating occasions and consumed more soft drinks and energy drinks, and the association was also seen in the regression analysis, although it did not reach statistical significance. Thus, they have a higher risk for dental caries and for being overweight and obesity-related diseases, including cardiovascular diseases [30], diabetes and cancers [1]. Dentists and dental hygienists should be aware of the rapidly exploding energy drink consumption; in the US, sales of energy drinks increased 60% from 2008 to 2012 [31]. Oral health professionals should inquire routinely if their patients regularly use energy drinks and inform them of the various health risks such as health risks related to caffeine content (hypertension, metabolic acidosis, convulsions, *etc.*), related to high-sugar content (obesity, dental caries) and related to acidic pH (dental erosion) [32].

### 4.3. The Role of Oral Health Professionals in Reducing Overweight Individuals

This study highlights the important role of oral health professionals in promoting a healthy lifestyle. The number of oral health professionals is important in Europe. A recent report states there are approximately 362,000 active dentists and 45,000 dental hygienists in the European Union (EU)/European Economic Area (EAA) [17]. Europeans aged 15 or over visit a dentist regularly, 57% of them had visited the dentist during the last year and only a minority (9%) of them had last visited a dentist five or more years ago [18]. 

Dentists and dental hygienists commonly see young adults who do not visit their primary care provider frequently. Thus, the dental community could provide a significant contribution to public health by endorsing weight management by healthy diet and when needed by referring their patients to dieticians or their general practitioner. Already, behavior interventions for tobacco cessation in dental offices have proven to be efficient and flexible support strategies and have positively influenced oral health professionals' knowledge of smoking and smoking cessation, their motivation to give advice, and their performance [33]. However, a recent literature review states that dietary advice is rarely provided by dentists, and, if it is provided, it is often quite unsatisfactory [34]. In order to change this, adequate nutrition education has to be incorporated into dental curriculum for dentists and dental hygienists and continuing education courses need to be provided on nutrition. Dental professionals need to seek information about the national nutrition policy and guidelines as well as how to give nutritional advice efficiently to their patients of different age groups. Innovative support strategies should be created in collaboration with experts in nutrition to help the oral health profession with this important work.

The risk factors for being overweight tend to cluster in individuals [11], and, in our study, those who were overweight exercised less than those who were non-overweight. Although oral health professionals are not experts in exercise counselling, it is important as part of promoting healthy life habits to inform the patients that lack of physical exercise is a risk factor for being overweight and many chronic diseases [8].

### 4.4. Limitations and Strengths

The cross-sectional design used gives limited power to illustrate causal relationships between oral health and lifestyle habits and being overweight. In addition, as this questionnaire study is part of a larger study, the number of dietary questions was limited and affected the choice of variables. Another limitation is that weight and height were self-reported. People have a tendency to underreport their weight and overreport their height [35]. If the BMI in this study were reported to be too low, it can be assumed that the associations would have been stronger with objectively measured values of weight and height. As the share of overweight persons increases with age, our results of young overweight women (16.5%) and men (34%) corresponds with the higher Western European average figures of 48% of overweight women and 61% of overweight men [36]. Our results concerning relationships between gender, education and being overweight are also in line with the European Commission Eurostat statistics on being overweight, stating that in all European Union Member States, the proportion of overweight men is considerably higher than women and that the share of overweight persons decreases as the educational level increases [37].

On the other hand, this study also has strengths. This study reports nationally representative data on six European countries and regionally representative on two European countries. The sampling method was quotas on sites. The patients who attended general dental practice were stratified by age, sex, profession and region in order to achieve an evenly distributed cross-section on young adult population in different participating countries. Furthermore, we provided new data about associations between oral health behavior and being overweight in an important age group.

## 5. Conclusions

To conclude, the overall prevalence of being overweight in the adult population aged 18–35 years from seven European countries (the Czech Republic, Estonia, Finland, France, Italy, Latvia and Spain) was 24.3%. In all participating countries, men reported higher values of being overweight. Overweight study participants had more contradictory oral health behavior than their thinner counterparts. The role of oral health professionals should be more active in endorsing weight management as they see young adults regularly.

## Figures and Tables

**Table 1:** Demographic characteristics and oral health behavior of study population according to BMI.

Variables	Normal or Underweight BMI < 25 (n = 2369) %	Overweight BMI ≥ 25 (n = 759) %	*p*-Value
**Age**			<0.001
18–25	44.2	30.6	
26–35	55.8	69.4	
**Sex**			<0.001
Male	39.7	62.8	
Female	60.3	37.2	
**Education**			0.001
To age 15	4.9	6.3	
To age 16–19	24.9	26.4	
To age 20+	43.9	48.2	
Still studying	26.3	19.1	
**Brushing frequency**			<0.001
1 per day	13.1	22.2	
≥2 per day	86.9	77.8	
**Toothbrush used**			0.750
Manual	77.2	78.3	
Electric	19.7	19.0	
**Fluoride toothpaste**			0.311
Yes	71.6	71.9	
No	9.1	7.1	
Do not know	19.3	20.8	
**Last dental visit**			0.016
0–2 years ago	86.8	81.6	
More than 2 years ago	13.2	18.4	
**Reason for last dental visit**			<0.001
Examination	61.5	52.6	
Routine dental treatment	24.6	26.2	
Emergency treatment	13.9	21.3	

**Table 2:** Comparison of lifestyle factors (smoking, exercise, and eating habits) among underweight or normal weight and overweight young adults.

Variables	Normal or Underweight (n = 2369) %	Overweight (n = 759) %	*p*-Value
**Smoking**			0.021
Often or occasionally	30.1	34.2	
Rarely or never	69.9	65.8	
**Exercise**			0.005
≥3 times a week	31.4	28.6	
1 to 2 times a week	34.0	30.2	
Less often	34.6	41.1	
**Consumption of soft drinks**			0.005
Often or occasionally	55.8	61.3	
Rarely or never	44.2	38.7	
**Consumption of energy drinks**			0.006
Often or occasionally	19.0	23.4	
Rarely or never	81.0	76.6	
**Consumption of fruit**			0.246
Often or occasionally	82.4	81.2	
Rarely or never	17.6	18.8	
**Consumption of dairy products**			0.279
Often or occasionally	90.7	89.9	
Rarely or never	9.3	10.1	
**Eating or drinking occasions**			<0.001
0–6 per day	81.9	76.3	
7 or more	18.1	23.7	

**Table 3:** Relationship of overweight to demographic, oral hygiene, dietary factors and exercise. Overweight patients (n = 759) are compared with normal or underweight patients (n = 2369). Odds ratios and 95% confidence limits based on binary logistic regression.

Independent Variables	OR (95% CI)	*p*-value
**Age**		
26–35	1.8 (1.5,2.1)	<0.001
18–25	Reference	
**Sex**		
Male	2.6 (2.2,3.0)	<0.001
Female	Reference	
**Education**		
Stopped studying before age of 20	1.0 (0.9,1.3)	0.640
Studied until age of 20 or is still studying	Reference	
**Brushing frequency**		
1 per day	1.6(1.3,2.0)	<0.001
≥2 per day	Reference	
**Last dental visit**		
More than 2 years ago	1.3 (1.1,1.7)	0.012
0–2 years ago	Reference	
**Reason for last dental visit**		
Emergency	1.6 (1.3,1.9)	<0.001
Other	Reference	
**Eating or drinking occasions**		
7 or more	1.4(1.1,1.7)	0.002
0–6 per day	Reference	
**Smoking**		
Often or occasionally	1.0 (0.9,1.0)	0.947
Rarely or never	Reference	
**Consumption of soft drinks**		
Often or occasionally	1.1 (0.9,1.3)	0.149
Rarely or never	Reference	
**Consumption of energy drinks**		
Often or occasionally	1.1(0.9,1.4)	0.224
Rarely or never	Reference	
**Exercise**		
<3 times a week	1.3(1.1,1.5)	0.014
≥3 times a week	Reference	
